# Migraine Symptom Progression During Pregnancy and Associations with Fetal Growth Patterns and Placental-Mediated Complications in Women with Pre-Existing Migraine: A Prospective Cohort Study

**DOI:** 10.3390/biomedicines14081655

**Published:** 2026-07-23

**Authors:** Milan Lackovic, Dejan Nikolic, Jovana Kuzmanovic Pficer, Sladjana Mihajlovic

**Affiliations:** 1 Harris Birthright Research Centre for Fetal Medicine, King’s College Hospital, London SE5 8BB, UK; milan.lackovic@nhs.net; 2 Department of Obstetrics and Gynecology, University Hospital “Dragiša Mišović”, 11000 Belgrade, Serbia; 3 Department of Physical Medicine and Rehabilitation, University Children’s Hospital, 11000 Belgrade, Serbia; denikol27@gmail.com; 4 Faculty of Medicine, University of Belgrade, 11000 Belgrade, Serbia; 5 Department of Medical Statistics and Informatics, Faculty of Dentistry, University of Belgrade, 11000 Belgrade, Serbia; jovana57@yahoo.com

**Keywords:** migraine, pregnancy, fetal growth restriction, placental dysfunction, pregnancy induced hypertension, gestational weight gain, preterm birth

## Abstract

**Background:** Migraine has been associated with vascular dysfunction and adverse pregnancy outcomes. However, the relationship between changes in migraine manifestations during pregnancy and fetal growth remains incompletely understood. This prospective study aimed to investigate whether migraine symptom progression during pregnancy is associated with abnormal fetal growth patterns and placental-mediated pregnancy complications among women with pre-existing migraine. **Methods:** This prospective cohort study included 400 pregnant women with a pre-pregnancy diagnosis of migraine who received antenatal care at the Department of Obstetrics and Gynecology, University Hospital “Dr Dragiša Mišović”, Belgrade, Serbia, between 2024 and 2026. Maternal migraine characteristics, pregnancy complications, and fetal growth assessments were evaluated. Fetal growth was categorized according to estimated fetal weight percentiles adjusted for gestational age. Univariable and multivariable logistic regression analyses were performed to identify factors associated with abnormal fetal growth. Because of the relatively small number of events, Firth’s penalized logistic regression was additionally performed as a sensitivity analysis. **Results:** Persistent or worsening migraine symptoms during pregnancy were more frequently observed among pregnancies complicated by impaired fetal growth, pregnancy-induced hypertension, gestational diabetes mellitus, abnormal gestational weight gain, and preterm birth. In univariable analyses, migraine worsening was associated with increased odds of abnormal fetal growth. However, after adjustment for maternal and obstetric factors, migraine worsening was no longer independently associated with abnormal fetal growth. Pregnancy-induced hypertension and inadequate gestational weight gain remained the strongest independent predictors. Findings were confirmed in sensitivity analyses using Firth’s penalized logistic regression. **Conclusions:** Among women with pre-existing migraine, persistent or worsening migraine manifestations during pregnancy were associated with a higher burden of placental-mediated complications and abnormal fetal growth patterns in unadjusted analyses. However, migraine worsening was not independently associated with abnormal fetal growth after adjustment for maternal and obstetric factors, suggesting that migraine symptom progression may represent a clinical marker of shared vascular, inflammatory, endothelial, and metabolic processes rather than an independent causal factor. Further prospective multicenter studies incorporating non-migraine control groups and detailed migraine phenotyping are warranted.

## 1. Introduction

Migraine is a chronic neurological disorder affecting millions of women and has increasingly been recognized as a systemic vascular disorder rather than solely a primary headache condition. Beyond its established association with long-term cardiovascular morbidity, migraine significantly impairs occupational performance, social functioning, and emotional well-being in women of reproductive age [1,2,3]. Increasing evidence suggests that migraine may also be associated with adverse pregnancy outcomes, underscoring its relevance as a maternal condition warranting closer clinical attention during pregnancy [4,5,6].

Pregnant women with migraine appear to be at increased risk for a range of maternal complications, including gestational hypertension and gestational diabetes mellitus, compared with the general obstetric population [7,8]. The coexistence of migraine with other chronic medical conditions may exert additive or synergistic effects, thereby amplifying the risk of unfavorable pregnancy outcomes and increasing vulnerability to both maternal and fetal complications [4,5]. Despite growing interest, the directionality of the association between migraine and comorbid chronic conditions remains incompletely understood, and whether migraine serves as an independent risk factor or reflects shared pathophysiological mechanisms continues to represent an important area of investigation [9,10].

The interaction between migraine and hormonal fluctuations is well established. Variations in estrogen and progesterone levels, particularly estrogen withdrawal, are recognized triggers for migraine attacks and symptom exacerbation [11]. Pregnancy is characterized by profound endocrine adaptations, creating a physiological environment that may substantially alter migraine manifestations. Evidence from menstrual cycle-related migraine supports the concept that sex hormone dynamics strongly influence migraine expression in women of reproductive age [11,12]. During pregnancy, sustained elevations in progesterone and relative stabilization of estrogen concentrations during the second and third trimesters are frequently associated with clinical improvement in migraine symptoms, whereas early gestational hormonal shifts may provoke symptom worsening in susceptible individuals [13,14]. These hormonal influences suggest that migraine during pregnancy reflects complex neurovascular and endocrine interactions that may extend beyond maternal symptom burden and potentially affect placental and fetal physiology.

Fetal growth is a key indicator of intrauterine well-being and an important determinant of perinatal morbidity and mortality. Serial assessment of fetal growth using standardized growth charts remains the cornerstone of fetal surveillance, guiding clinical decisions regarding pregnancy monitoring, timing, and mode of delivery [15]. Deviations from expected growth trajectories are associated with adverse neonatal and long-term health outcomes, emphasizing the importance of identifying maternal conditions that may influence fetal growth patterns [16,17].

While hypertensive disorders of pregnancy, diabetes mellitus, obesity, and chronic maternal cardiovascular, renal, and hepatic diseases are well-recognized risk factors requiring enhanced surveillance [18,19,20,21], migraine remains relatively underrecognized as a potential contributor to altered fetal growth unless accompanied by severe neurological manifestations [4,6]. This gap in clinical awareness may partly reflect the lack of objective biomarkers and standardized metrics to quantify the impact of migraine during pregnancy. Nevertheless, growing evidence linking migraine to vascular dysfunction, inflammation, and endothelial impairment supports the hypothesis that migraine may be associated with altered placental function and fetal growth dynamics [4,9,19].

Most available studies have evaluated pregnancy outcomes according to the presence or absence of migraine before pregnancy. Far fewer investigations have examined whether changes in migraine manifestations during gestation carry additional prognostic information regarding placental function and fetal growth. Because migraine symptoms often improve during pregnancy whereas others persist or worsen, symptom evolution may represent a clinically relevant marker of maternal vascular adaptation rather than migraine severity alone. Therefore, the primary objective of this prospective cohort study was to investigate whether changes in migraine manifestations during pregnancy are associated with abnormal fetal growth patterns among women with pre-existing migraine. Specifically, the study sought to evaluate the relationship between migraine manifestations during pregnancy and fetal growth trajectories assessed by standardized fetal biometric measurements and growth percentiles. A secondary objective was to evaluate the relationship between migraine symptom progression and placental-mediated pregnancy complications, including pregnancy-induced hypertension, gestational diabetes mellitus, abnormal gestational weight gain, and preterm birth. We further explored whether migraine worsening remained independently associated with abnormal fetal growth after adjustment for relevant maternal and obstetric characteristics.

## 2. Methods

### 2.1. Study Design and Population

This prospective cohort study included 400 pregnant women receiving antenatal care at the Department of Obstetrics and Gynecology, University Hospital “Dr Dragiša Mišović”, Belgrade, Serbia, between 2024 and 2026. The study was approved by the Institutional Review Board of the University Hospital “Dr Dragiša Mišović” (Approval No. 11297/3; 27 May 2024). Pregnancies were eligible for inclusion if complete maternal clinical data and serial fetal growth assessments were available throughout pregnancy.

**Figure 1:**
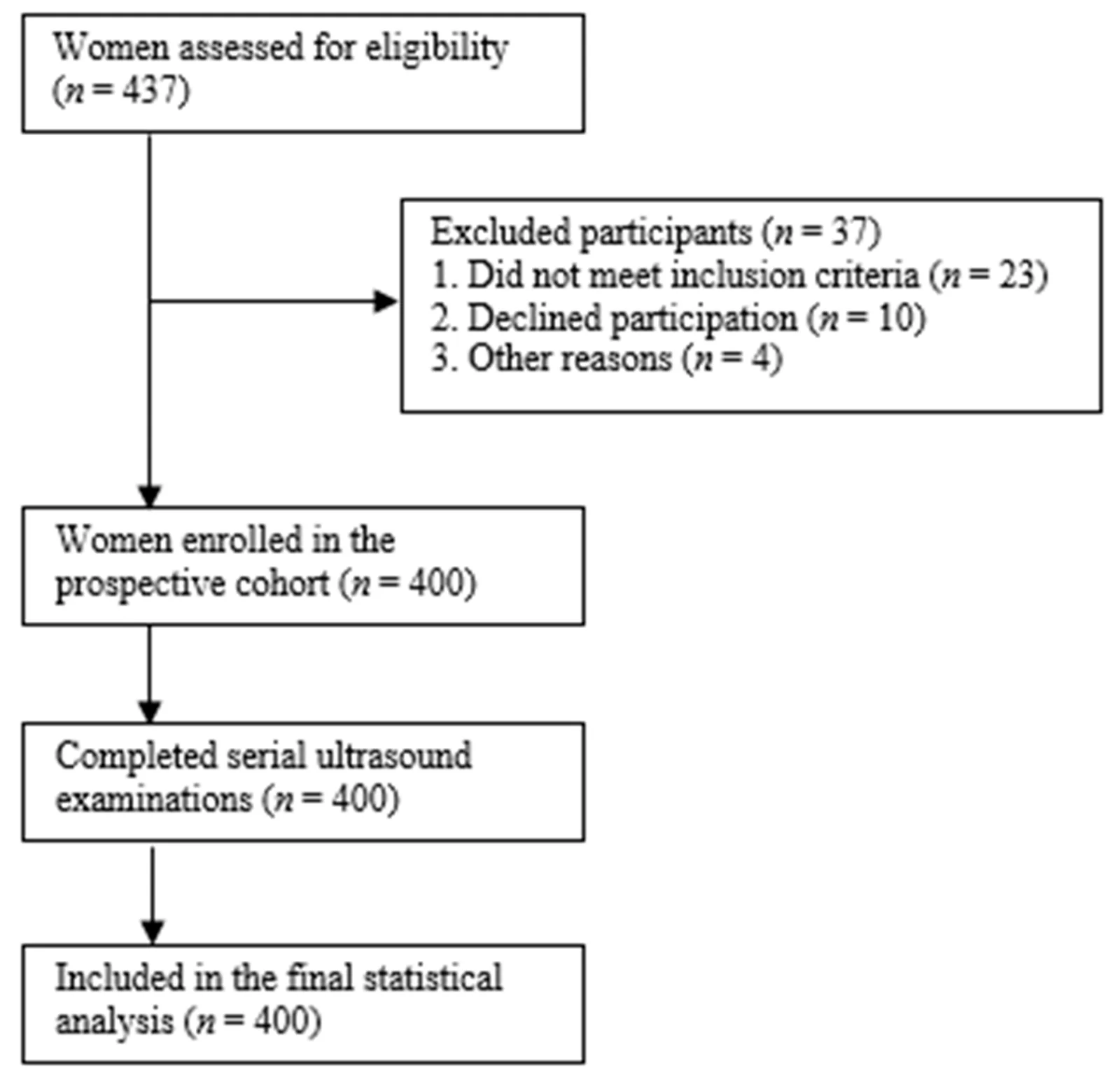
Flow chart. Study participants’ inclusion.

All participants had a documented diagnosis of migraine prior to pregnancy according to the International Classification of Headache Disorders, 3rd edition (ICHD-3) criteria [22]. Women with multiple gestations, known fetal structural or chromosomal anomalies, pre-existing maternal conditions known to affect fetal growth (including chronic hypertension, pregestational diabetes mellitus, renal disease, and autoimmune disorders), or incomplete fetal growth data were excluded. Study participants’ inclusion is presented in flow chart (Figure 1).

### 2.2. Exposure Variables and Covariates

Because all participants had a pre-pregnancy diagnosis of migraine, the primary exposure variables were migraine characteristics during pregnancy, including migraine frequency, intensity, and symptom progression. Maternal characteristics and potential confounding variables included advanced maternal age (>35 years), parity, pre-pregnancy obesity (defined as body mass index [BMI] > 30 kg/m^2^), gestational weight gain (GWG), pregnancy-induced hypertension (PIH), gestational diabetes mellitus (GDM), labor induction, and preterm birth.

According to the Institute of Medicine recommendations [23], GWG was categorized as insufficient, adequate, or excessive. Diagnosis of GDM was established according to the American Diabetes Association guidelines [24]. PIH included gestational hypertension, preeclampsia, eclampsia, and HELLP syndrome.

Migraine manifestations during pregnancy were assessed using structured questionnaires evaluating migraine frequency and intensity before and during pregnancy. Pre-pregnancy migraine characteristics were assessed retrospectively at enrollment. Questionnaires were completed at two time points: during the first antenatal visit and within three months postpartum. Migraine symptom patterns during pregnancy were categorized as decreased, unchanged, or increased compared with the pre-pregnancy period. None of the participants reported using vasoactive medications for the treatment of migraine during pregnancy.

### 2.3. Fetal Growth Assessment

Fetal growth was assessed using standardized ultrasound biometric measurements, including biparietal diameter, head circumference, abdominal circumference, and femur length, obtained during routine antenatal follow-up according to institutional protocols. Estimated fetal weight (EFW) was calculated using the Hadlock formula incorporating standard fetal biometric parameters and expressed as gestational age-adjusted percentiles according to established fetal growth charts. All ultrasound examinations were performed according to standardized institutional protocols by experienced obstetricians trained in fetal ultrasound assessment.

Fetal growth between the 10th and 90th percentile was classified as appropriate for gestational age (AGA). Fetuses with EFW below the 10th percentile in the absence of abnormal Doppler findings were classified as small for gestational age (SGA). Fetuses with EFW below the 10th percentile and abnormal Doppler findings suggestive of placental insufficiency were classified as fetal growth restriction (FGR) [16]. Fetal growth above the 90th percentile was classified as large for gestational age (LGA).

Participants were categorized into five predefined fetal growth groups according to longitudinal fetal growth assessments and gestational age-adjusted EFW percentiles. For additional analyses, participants were further classified into two broader groups: normal fetal growth and abnormal fetal growth. Abnormal fetal growth was defined as EFW below the 10th percentile or above the 90th percentile for gestational age. Although these categories may reflect different biological mechanisms, both represent clinically relevant deviations from expected fetal growth patterns.

### 2.4. Outcome Measures

The primary outcome was fetal growth category based on gestational age-adjusted EFW percentiles. Secondary outcomes included changes in migraine frequency and intensity during pregnancy compared with the pre-pregnancy period, incidence of SGA, FGR, and LGA, PIH, GDM, induction of labor, preterm birth, obesity, GWG distribution and advanced maternal age.

### 2.5. Statistical Analysis

Statistical analyses were performed using IBM SPSS Statistics version 22 (IBM Corp., Armonk, NY, USA). Continuous variables are presented as median values with minimum and maximum ranges, whereas categorical variables are presented as frequencies and percentages. Differences among fetal growth groups were assessed using the Kruskal–Wallis test for continuous variables and the Pearson χ^2^ test for categorical variables. For regression analyses, participants were additionally classified into two groups according to fetal growth status: normal fetal growth (EFW between the 10th and 90th percentiles for gestational age) and abnormal fetal growth (EFW below the 10th percentile or above the 90th percentile for gestational age). Univariable logistic regression analyses were initially performed to evaluate associations between maternal demographic characteristics, obstetric factors, migraine-related variables, and abnormal fetal growth. Variables demonstrating the strongest associations with abnormal fetal growth in univariable analyses were subsequently entered into a multivariable logistic regression model to identify factors independently associated with the outcome. Results are presented as odds ratios (ORs) with corresponding 95% confidence intervals (CIs).

To identify factors associated with abnormal fetal growth, binary logistic regression analysis was performed. Initially, univariate analyses were conducted, followed by a multivariable logistic regression model using the Enter method. The results are presented as odds ratios (ORs) with 95% confidence intervals (95% CIs), and statistical significance was defined as *p* < 0.05.

Given the relatively small number of events, an additional sensitivity analysis was performed using Firth’s penalized logistic regression in the R statistical environment version 4.5.1 https://cran.r-project.org/bin/windows/base/old/4.5.1/ (accessed on 1 August 2025), employing the logistf package version 1.26.1 https://cran.r-project.org/web/packages/logistf/index.html (accessed on 1 August 2025). The results of the Firth regression are presented as ORs with 95% CIs.

Because changes in migraine frequency and intensity showed a strong correlation, a new binary variable, “migraine worsening,” was created and defined as the presence of worsening in migraine frequency and/or intensity during pregnancy compared with the pre-pregnancy period. This variable was used in the multivariable models to avoid instability of the estimates resulting from the correlation between individual migraine characteristics.

All statistical tests were two-sided, and *p*-values < 0.05 were considered statistically significant.

## 3. Results

**Figure 2:**
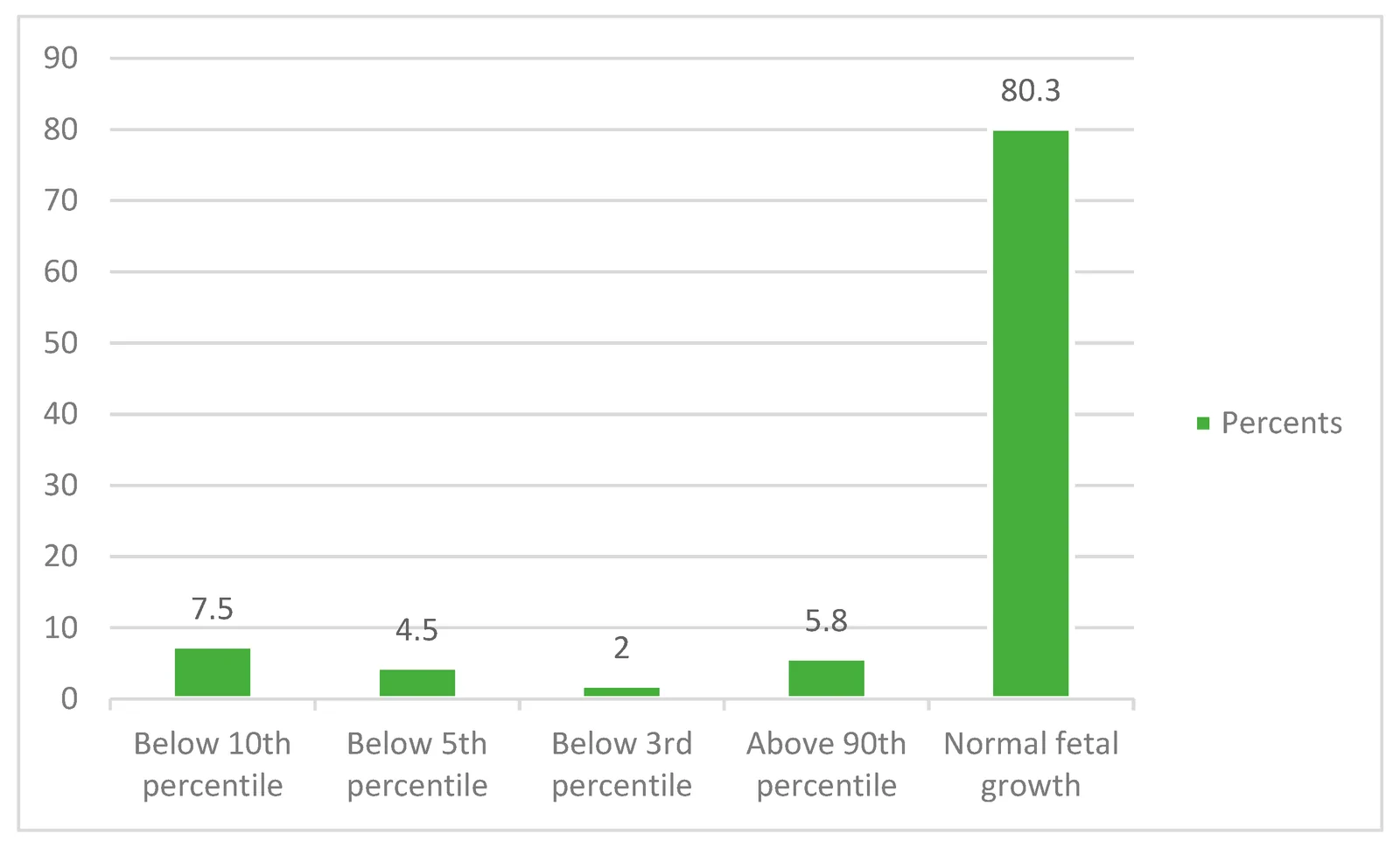
Distribution of fetal growth categories among the study population.

Among the 400 study participants, most fetuses demonstrated normal intrauterine growth patterns. Specifically, 321 fetuses (80.3%) had estimated fetal weight between the 10th and 90th percentiles and were classified as appropriate for gestational age. Abnormal fetal growth patterns were identified in the remaining pregnancies. Thirty fetuses (7.5%) had EFW between the 5th and 10th percentiles, 18 (4.5%) between the 3rd and 5th percentiles, and 8 fetuses (2.0%) demonstrated severe fetal growth restriction with EFW below the 3rd percentile. In contrast, 23 fetuses (5.8%) had EFW above the 90th percentile and were classified as large for gestational age (Figure 2).

Table 1 summarizes maternal, obstetric, and migraine-related characteristics according to fetal growth categories. Significant differences between fetal growth groups were observed for several maternal and pregnancy-related variables. Maternal age did not differ significantly across fetal growth categories (*p* = 0.173). However, parity differed significantly between groups (*p* = 0.014), with women carrying normally grown fetuses demonstrating higher median parity compared with pregnancies complicated by abnormal fetal growth. Maternal obesity and gestational weight gain demonstrated strong associations with fetal growth patterns. Obesity was particularly prevalent among women carrying fetuses below the 5th percentile, where two-thirds of subjects had BMI > 30 kg/m^2^ (*p* = 0.020). Significant differences were also observed regarding gestational weight gain distribution (*p* < 0.001). Excessive gestational weight gain predominated among pregnancies with fetal growth above the 90th percentile, whereas insufficient gestational weight gain was substantially more frequent among pregnancies complicated by fetal growth restriction. The occurrence of pregnancy-induced hypertension differed markedly across fetal growth groups (*p* < 0.001). PIH was present in all women carrying fetuses below the 5th percentile and in 87.5% of pregnancies with fetal growth below the 3rd percentile, compared with only 7.2% of pregnancies with normal fetal growth. Similarly, gestational diabetes mellitus was significantly more common among pregnancies with abnormal fetal growth (*p* < 0.001), particularly in the group with fetal growth above the 90th percentile.

Migraine manifestations during pregnancy differed significantly between fetal growth groups. Worsening migraine intensity during pregnancy was substantially more frequent among women carrying fetuses below the 5th and 3rd percentiles, reported in 77.8% and 87.5% of cases, respectively (*p* = 0.001). Likewise, increased migraine frequency during pregnancy was significantly more common in these groups compared with pregnancies demonstrating normal fetal growth (*p* = 0.021).

Pregnancies complicated by impaired fetal growth were also characterized by substantially higher rates of preterm birth (*p* < 0.001). Preterm delivery occurred in 94.4% of pregnancies with fetal growth below the 5th percentile and in 87.5% of those below the 3rd percentile, compared with only 5.9% among pregnancies with normal fetal growth. Delivery mode also differed significantly between groups (*p* < 0.001). Induction of labor was substantially more frequent among pregnancies complicated by fetal macrosomia, whereas spontaneous delivery predominated among normally grown fetuses.

As expected, fetal growth restriction and large for gestational age distributions were strongly associated with fetal growth categories (both *p* < 0.001), confirming the validity of the fetal growth classification used in the study.

In Table 2, multivalent logistic regression analysis of predictors of abnormal fetal growth was presented. For the purposes of regression analysis study participants were additionally classified into two broader groups according to fetal growth status: normal fetal growth and abnormal fetal growth. Abnormal fetal growth was defined as EFW below the 10th percentile or above the 90th percentile for gestational age.

A univariable logistic regression analysis was initially performed to assess the association between potential predictors and fetal growth. The variables examined included maternal age, parity, body mass index (BMI), gestational weight gain (GWG), pregnancy-induced hypertension (PIH), gestational diabetes mellitus (GDM), headache intensity, headache frequency and preterm birth. Based on the univariable analysis, five variables demonstrating the strongest associations with the outcome were selected and subsequently included in the multivariable logistic regression model.

Following the previous analysis, in the multivariable logistic regression analysis showed that PIH, GDM, insufficient GWG, and preterm birth were significantly associated with the outcome.

Although migraine worsening was associated with abnormal fetal growth in the univariable analyses, this association disappeared after adjustment for pregnancy-induced hypertension and gestational weight gain, suggesting that the observed unadjusted association may largely reflect shared vascular or metabolic characteristics rather than an independent effect of migraine symptom progression.

The presence of PIH was associated with a more than 5-fold increase in the odds of the outcome (OR = 5.578; 95% CI: 2.238–13.901; *p* < 0.001). Similarly, GDM was associated with approximately three times higher odds of the outcome (OR = 3.266; 95% CI: 1.563–6.827; *p* = 0.002).

Compared to normal GWG, insufficient GWG had about threefold increased odds (OR = 3.049; 95% CI: 1.005–9.251; *p* = 0.049), while excessive GWG was not significantly associated (OR = 1.381; 95% CI: 0.657–2.905; *p* = 0.395).

Preterm birth was also a significant predictor, with more than fourfold higher odds of the outcome compared to full-term birth (OR = 4.198; 95% CI: 1.704–10.339; *p* = 0.002). Parity was not a statistically significant predictor in this model when comparing multiparous to nulliparous women (OR = 0.693; 95% CI: 0.415–1.156; *p* = 0.160) (Table 3). The multivariable logistic regression model was statistically significant (Omnibus test: χ^2^(5) = 138.593, *p* < 0.001), indicating that the set of predictors as a whole reliably distinguished between normal and abnormal fetal growth.

To provide greater clinical and biological insight, logistic regression analyses were performed separately for fetuses with an estimated fetal weight (EFW) below the 10th percentile and those with an EFW above the 90th percentile. Table 3 presents the analyses comparing pregnancies with fetal growth below the 10th percentile with pregnancies in which fetal growth was appropriate for gestational age.

In the univariable logistic regression analyses, maternal age was not significantly associated with fetal growth below the 10th percentile (OR = 1.031; 95% CI: 0.986–1.079; *p* = 0.185), whereas higher parity was associated with lower odds of the outcome (OR = 0.448; 95% CI: 0.268–0.749; *p* = 0.002). Conversely, higher pre-pregnancy BMI was associated with increased odds of fetal growth below the 10th percentile (OR = 1.879; 95% CI: 1.057–3.342; *p* = 0.032).

Gestational weight gain (GWG) category was significantly associated with fetal growth below the 10th percentile. Compared with adequate GWG, excessive GWG was associated with approximately 2.8-fold higher odds of the outcome (OR = 2.818; 95% CI: 1.412–5.626; *p* = 0.003), whereas inadequate GWG demonstrated an even stronger association (OR = 20.304; 95% CI: 8.463–48.714; *p* < 0.001). Pregnancy-induced hypertension (PIH) (OR = 38.870; 95% CI: 18.568–81.367; *p* < 0.001) and gestational diabetes mellitus (GDM) (OR = 3.272; 95% CI: 1.774–6.033; *p* < 0.001) were also significantly associated with fetal growth below the 10th percentile.

Among the migraine-related characteristics, worsening migraine frequency was associated with increased odds of fetal growth below the 10th percentile (OR = 2.201; 95% CI: 1.051–4.609; *p* = 0.036), while changes in migraine intensity demonstrated a significant overall association (*p* = 0.025). Because worsening migraine frequency and intensity showed substantial collinearity, a composite binary variable, migraine worsening, defined as worsening of migraine frequency and/or intensity during pregnancy compared with the pre-pregnancy period, was created. In the univariable analysis, migraine worsening was associated with a 1.8-fold increase in the odds of fetal growth below the 10th percentile (OR = 1.775; 95% CI: 1.002–3.145; *p* = 0.049).

In the multivariable logistic regression model (Omnibus test: χ^2^ = 128.302, df = 7; *p* < 0.001), migraine worsening was no longer independently associated with fetal growth below the 10th percentile after adjustment for maternal and obstetric characteristics (OR = 0.728; 95% CI: 0.300–1.768; *p* = 0.483). In contrast, pregnancy-induced hypertension remained the strongest independent predictor, conferring an approximately 26-fold increase in the odds of fetal growth below the 10th percentile (OR = 25.864; 95% CI: 11.102–60.256; *p* < 0.001). Inadequate gestational weight gain also remained independently associated with the outcome (OR = 3.681; 95% CI: 1.118–12.116; *p* = 0.032), whereas none of the remaining variables retained statistical significance.

Because of the relatively small number of pregnancies with fetal growth below the 10th percentile (*n* = 56), Firth’s penalized logistic regression was performed as a sensitivity analysis to reduce small-sample bias. The results closely mirrored those of the conventional multivariable model, supporting the robustness of the primary findings. Migraine worsening remained unrelated to fetal growth below the 10th percentile (OR = 0.74; 95% CI: 0.32–1.73; *p* = 0.495), whereas pregnancy-induced hypertension remained the strongest independent predictor (OR = 21.57; 95% CI: 9.80–50.37; *p* < 0.001). Inadequate gestational weight gain also remained independently associated with fetal growth below the 10th percentile (OR = 3.34; 95% CI: 1.06–10.76; *p* = 0.040), confirming the stability of the multivariable analysis (Table 3).

## 4. Discussion

Previous large population-based studies have demonstrated that women with migraine are at increased risk of adverse pregnancy outcomes, including hypertensive disorders of pregnancy and low birth weight [25]. However, the mechanisms linking migraine and fetal growth abnormalities remain incompletely understood. Our findings contribute to this field by suggesting that the association between migraine and adverse fetal growth may be largely mediated by maternal vascular and metabolic factors rather than by migraine itself. In our cohort, 80.3% of fetuses demonstrated growth patterns between the 10th and 90th percentiles, consistent with expected population-based fetal growth distributions, where approximately 80% of fetuses are expected to fall within this range by definition [26]. Although the overall proportion of fetuses with EFW below the 10th percentile was not substantially different from expected population distributions, an important observation emerged following Doppler assessment. Among fetuses initially classified between the 5th and 10th percentile, only two fulfilled criteria for constitutionally small-for-gestational-age fetuses, whereas the majority demonstrated Doppler features consistent with fetal growth restriction. These findings indicate that placental dysfunction and impaired uteroplacental perfusion, rather than constitutional fetal smallness alone, likely contributed to impaired fetal growth in this population.

The disappearance of the association between migraine worsening and abnormal fetal growth after multivariable adjustment represents an important finding. Migraine symptoms frequently improve during pregnancy, with previous studies reporting remission or substantial improvement in approximately 50–75% of affected women [27]. In contrast, in our cohort, worsening migraine intensity was reported in 77.8% and 87.5% of women carrying fetuses below the 5th and 3rd percentiles, respectively, while more than two-thirds also experienced increased migraine frequency during pregnancy. Notably, none of the participants in growth-restricted groups reported unchanged migraine manifestations. This observation suggests that persistent migraine activity may identify women with increased biological susceptibility to pregnancy complications, even though migraine progression itself does not appear to independently determine fetal growth after accounting for relevant maternal factors.

A plausible explanation is that migraine and placental-mediated pregnancy complications represent overlapping manifestations of shared vascular, inflammatory, and metabolic disturbances. Endothelial dysfunction, systemic inflammation, oxidative stress, vascular hyperreactivity, and prothrombotic tendencies have all been implicated in migraine pathophysiology and may adversely affect uteroplacental perfusion, spiral artery remodeling, placental angiogenesis, and overall placental function [28,29,30]. Therefore, worsening migraine symptoms during pregnancy may serve as a clinical marker of underlying vascular vulnerability rather than a direct cause of placental dysfunction. Reverse causality should also be considered when interpreting these findings. Rather than migraine symptom progression contributing directly to placental dysfunction, evolving placental insufficiency or maternal vascular maladaptation may itself contribute to persistence or worsening of migraine during pregnancy. Because migraine manifestations and obstetric complications evolved throughout gestation, the temporal sequence between these events cannot be established with certainty. Therefore, the observed associations should be interpreted as evidence of shared susceptibility pathways rather than evidence of a direct causal relationship.

Pregnancy-induced hypertension emerged as the strongest independent predictor of impaired fetal growth in both conventional and Firth regression analyses. More than half of women carrying fetuses below the 10th percentile, and nearly all women carrying fetuses below the 5th percentile, ultimately developed PIH. This finding is biologically plausible, as hypertensive disorders of pregnancy are closely linked to impaired trophoblast invasion, inadequate spiral artery remodeling, and reduced uteroplacental perfusion, all of which represent fundamental mechanisms underlying placental insufficiency and fetal growth restriction [31]. The persistence of PIH as an independent predictor after multivariable adjustment emphasizes the dominant role of placental vascular dysfunction in determining fetal growth trajectories.

The close association between migraine and PIH has been consistently reported in previous studies, with women affected by migraine demonstrating an increased risk of developing hypertensive disorders during pregnancy, particularly when migraine symptoms fail to improve with advancing gestation [32,33]. Whether PIH represents a downstream consequence of the same vascular susceptibility underlying migraine or an independent mediator contributing to adverse pregnancy outcomes remains uncertain [4,8]. Nevertheless, our findings suggest that migraine, PIH, and fetal growth restriction are more likely interconnected manifestations of impaired maternal cardiovascular adaptation than isolated disease entities. Rather than acting through independent mechanisms, these conditions probably share overlapping pathways involving endothelial dysfunction, vascular hyperreactivity, inflammation, and oxidative stress.

The placenta may represent the central biological link connecting these observations. Successful pregnancy requires profound maternal cardiovascular, endocrine, metabolic, and immunological adaptations that enable adequate placental development and establish low-resistance uteroplacental circulation [34,35]. Simultaneously, the placenta becomes a major endocrine organ producing increasing concentrations of estrogen, progesterone, neurosteroids, endogenous opioids, and numerous vasoactive mediators throughout gestation. These physiological adaptations are believed to contribute substantially to the improvement or complete remission of migraine experienced by most women during pregnancy [36]. Failure to establish these adaptive mechanisms may therefore explain why a subgroup of women continues to experience persistent or worsening migraine while simultaneously developing placental-mediated complications such as PIH, FGR, and ultimately preterm birth. Within this framework, persistent migraine during pregnancy may be viewed less as an isolated neurological phenomenon than as a clinical manifestation of inadequate maternal adaptation to pregnancy.

The direct relationship between migraine and fetal growth nevertheless remains difficult to isolate, particularly given the presence of multiple vascular and metabolic comorbidities frequently associated with migraine. The contribution of maternal metabolic status further supports this hypothesis. Obesity is increasingly recognized as a chronic inflammatory and vascular disorder characterized by endothelial dysfunction, oxidative stress, metabolic dysregulation, and persistent low-grade inflammation, mechanisms that substantially overlap with those implicated in both migraine pathophysiology and placental dysfunction [37,38]. In our cohort, obesity affected more than one-third of participants and was particularly common among pregnancies complicated by impaired fetal growth. These findings are consistent with previous meta-analyses [37] demonstrating a significant association between obesity and migraine, particularly chronic and high-frequency migraine, suggesting that both conditions may reflect common disturbances in vascular and metabolic homeostasis.

Interestingly, pre-pregnancy obesity alone was not sufficient to explain the observed differences in fetal growth. Women carrying fetuses below the 3rd percentile exhibited one of the lowest rates of obesity but demonstrated marked disturbances in gestational weight gain, with nearly equal proportions experiencing either insufficient or excessive weight gain. In contrast, women carrying fetuses above the 90th percentile most frequently exceeded recommended gestational weight gain despite only moderately increased obesity prevalence. These observations suggest that maternal metabolic adaptation during pregnancy, rather than baseline anthropometric status alone, may exert a greater influence on fetal growth trajectories. This interpretation is reinforced by our multivariable analysis, in which inadequate gestational weight gain remained independently associated with impaired fetal growth, whereas pre-pregnancy obesity did not. Insufficient gestational weight gain has previously been associated with impaired placental development, reduced nutrient availability, fetal growth restriction, and earlier delivery [39,40,41]. Conversely, excessive gestational weight gain has consistently been linked to gestational diabetes mellitus (GDM), fetal overgrowth, and large-for-gestational-age infants [42]. Together, these findings suggest that dynamic maternal metabolic adaptation throughout pregnancy may be more relevant for fetal growth than maternal body mass index at conception.

Gestational diabetes mellitus also emerged as an important contributor to abnormal fetal growth in our cohort. Although GDM is traditionally associated with fetal overgrowth, it was also more prevalent among pregnancies complicated by fetal growth restriction. This apparent paradox likely reflects the complex interaction between maternal hyperglycemia, placental dysfunction, altered nutrient transport, and diabetes-associated microangiopathy. Depending on the balance between metabolic substrate availability and placental vascular function, GDM may contribute to both excessive and impaired fetal growth [43,44]. These observations further emphasize that fetal growth is determined by the interaction between maternal metabolism and placental function rather than by isolated maternal characteristics.

Prematurity represented the final common adverse outcome of these interrelated pathological processes. Fetuses demonstrating abnormal growth patterns experienced substantially higher rates of preterm delivery than those with normal fetal growth, and almost all pregnancies complicated by severe fetal growth restriction required delivery before term. This finding most likely reflects contemporary obstetric management, in which delivery often becomes the only effective intervention once progressive placental insufficiency threatens fetal wellbeing. Rather than representing an isolated outcome, prematurity should therefore be considered the downstream consequence of progressive placental dysfunction, maternal vascular maladaptation, and fetal compromise.

The present prospective cohort study provides new insight into the relationship between migraine symptom evolution during pregnancy and fetal growth among women with pre-existing migraine. Although persistent or worsening migraine manifestations were associated with a higher burden of abnormal fetal growth and placental-mediated pregnancy complications in univariable analyses, this association was no longer significant after adjustment for maternal and obstetric factors. In contrast, PIH and insufficient GWG remained the strongest independent predictors of abnormal fetal growth. These findings suggest that migraine symptom progression may not represent an independent determinant of impaired fetal growth, but rather a clinical manifestation of a broader maternal vascular and metabolic phenotype associated with placental-mediated pregnancy complications. Taken together, our findings support a unifying biological model in which the placenta occupies a central role linking migraine symptom evolution, maternal vascular adaptation, metabolic regulation, and fetal growth. Women whose migraine improves during pregnancy may represent those who successfully undergo the complex endocrine and cardiovascular adaptations required for normal placentation. Conversely, persistent or worsening migraine may identify women in whom these adaptive mechanisms are incomplete, predisposing them to hypertensive disorders, abnormal gestational weight gain, placental insufficiency, fetal growth abnormalities, and ultimately preterm birth. Within this conceptual framework, migraine should not be viewed solely as a neurological disorder during pregnancy but rather as a potential clinical marker of impaired maternal adaptation and increased susceptibility to placental-mediated complications.

Several limitations should be acknowledged when interpreting our results. First, this was a single-center cohort study, which may limit the generalizability of the findings to broader obstetric populations and other ethnic or geographic groups. Additionally, all participants originated from the Serbian population, and regional differences in maternal characteristics, healthcare systems, and pregnancy management strategies may influence the external applicability of the results. Second, the study did not include a non-migraine control group. The absence of a non-migraine comparison group limits the ability to determine whether migraine itself independently contributes to abnormal fetal growth. Additionally, pre-pregnancy migraine characteristics were assessed retrospectively, introducing the possibility of recall bias. Information regarding migraine subtype, aura status, medication use during pregnancy, smoking status, and socioeconomic factors was not systematically collected and may have influenced the observed associations. Finally, some fetal growth subgroups contained relatively small numbers of participants, limiting statistical power.

## 5. Conclusions

Among women with pre-existing migraine, persistent or worsening migraine manifestations during pregnancy were associated with a higher burden of placental-mediated pregnancy complications and abnormal fetal growth in univariable analyses. However, these associations were no longer significant after adjustment for maternal and obstetric factors, whereas pregnancy-induced hypertension and insufficient gestational weight gain remained the strongest independent predictors of impaired fetal growth. These findings suggest that migraine symptom progression is unlikely to represent an independent determinant of fetal growth restriction but may instead reflect an underlying maternal vascular and metabolic phenotype associated with impaired placental adaptation.

Our results support the concept that migraine, hypertensive disorders of pregnancy, abnormal gestational weight gain, placental dysfunction and fetal growth restriction, represent interconnected manifestations of shared biological pathways rather than isolated clinical entities. Persistent migraine during pregnancy may therefore serve as an easily recognizable clinical marker of increased susceptibility to placental-mediated complications, identifying women who may benefit from enhanced antenatal surveillance.

## Figures and Tables

**Table 1:** Maternal, obstetric, and migraine-related characteristics according to fetal growth categories.

Tested Variables		Fetal Growth					*p*
		Below 10%	Below 5%	Below 3%	Above 90%	Normal	
Age, years (MV ± SD)		32.97 ± 4.80	32.33 ± 5.63	28.88 ± 6.29	30.09 ± 6.02	30.98 ± 6.33	0.173 *
Number of deliveries (Median, Minimum-Maximum)		1 (1–3)	1 (1–3)	1 (1–2)	1 (1–2)	2 (1–4)	0.014 *
Age above 35 years, *n* (%)	No	17 (56.7)	13 (72.2)	6 (75.0)	16 (69.6)	233 (72.6)	0.480 **
	Yes	13 (43.3)	5 (27.8)	2 (25.0)	7 (30.4)	88 (27.4)	
Body mass index, *n* (%)	<30	18 (60.0)	6 (33.3)	6 (75.0)	18 (78.3)	219 (68.4)	0.020 **
	>30	12 (40.0)	12 (66.7)	2 (25.0)	5 (21.7)	101 (31.6)	
Gestational weigh gain, *n* (%)	normal	9 (30.0)	6 (33.3)	2 (25.0)	8 (34.8)	218 (67.9)	<0.001 **
	above	13 (43.3)	4 (22.2)	3 (37.5)	14 (60.9)	91 (28.3)	
	bellow	8 (26.7)	8 (44.4)	3 (37.5)	1 (4.3)	12 (3.7)	
Pregnancy-induced hypertension, *n* (%)	No	13 (43.3)	0 (0.0)	1 (12.5)	18 (78.3)	298 (92.8)	<0.001 **
	Yes	17 (56.7)	18 (100.0)	7 (87.5)	5 (21.7)	23 (7.2)	
Gestational diabetes mellitus, *n* (%)	No	22 (73.3)	8 (44.4)	4 (50.0)	8 (34.8)	268 (83.5)	<0.001 **
	Yes	8 (26.7)	10 (55.6)	4 (50.0)	15 (65.2)	53 (16.5)	
Migraine pain intensity during pregnancy versus before pregnancy, *n* (%)	Decreased	8 (26.7)	4 (22.2)	1 (12.5)	8 (34.8)	92 (28.7)	0.001 **
	Unchanged	13 (43.3)	0 (0.0)	0 (0.0)	8 (34.8)	118 (36.8)	
	Increased	9 (30.0)	14 (77.8)	7 (87.5)	7 (30.4)	111 (34.6)	
Migraine frequency during pregnancy versus before pregnancy, *n* (%)	Decreased	8 (26.7)	4 (22.2)	1 (12.5)	8 (34.8)	93 (29.0)	0.021 **
	Unchanged	14 (46.7)	3 (16.7)	2 (25.0)	9 (39.1)	150 (46.7)	
	Increased	8 (26.7)	11 (61.1)	5 (62.5)	6 (26.1)	78 (24.3)	
Preterm delivery, *n* (%)	No	13 (43.3)	1 (5.6)	1 (12.5)	18 (78.3)	302 (94.1)	<0.001 **
	Yes	17 (56.7)	17 (94.4)	7 (87.5)	5 (21.7)	19 (5.9)	
Induction of labor, *n* (%)	No	16 (53.3)	10 (55.6)	5 (62.5)	5 (21.7)	248 (77.3)	<0.001 **
	Yes	14 (46.7)	8 (44.4)	3 (37.5)	18 (78.3)	73 (22.7)	
Fetal growth restriction, *n* (%)	No	2 (6.7)	0 (0.0)	0 (0.0)	23 (100.0)	321 (100.0)	<0.001 **
	Yes	28 (93.3)	18 (100.0)	8 (100.0)	0 (0.0)	0 (0.0)	
Large for gestational age, *n* (%)	No	29 (96.7)	18 (100.0)	8 (100)	0 (0.0)	321 (100.0)	<0.001 **
	Yes	1 (3.3)	0 (0.0)	0 (0.0)	23 (100.0)	0 (0.0)	

*, Kruskal–Wallis test; **, Pearson Chi-Square test; MV, mean value; SD, standard deviation.

**Table 2:** Multivalent logistic regression analysis of predictors of abnormal fetal growth.

Tested Variables	B	*p*	OR	95% CI for OR	
				Lower	Upper
Number of deliveries	−0.367	0.160	0.693	0.415	1.156
Pregnancy-induced hypertension	1.719	<0.001	5.578	2.238	13.901
Gestational diabetes mellitus	1.184	0.002	3.266	1.563	6.827
Adequate gestational weigh gain		0.139			
Excessive gestational weigh gain	0.323	0.395	1.381	0.657	2.905
Insufficient gestational weigh gain	1.115	0.049	3.049	1.005	9.251
Preterm delivery	1.435	0.002	4.198	1.704	10.339

B, regression coefficient; *p*, statistical significance value; OR, odds ratio; CI, confidence interval.

**Table 3:** Univariable and multivariable logistic regression analyses of factors associated with estimated fetal weight below the 10th percentile.

Tested Variables	B	S.E.	Wald	*p*	OR	95% CI for OR	
						Lower	Upper
Number of deliveries	−0.382	0.322	1.402	0.236	0.683	0.363	1.284
Body mass index	−0.144	0.422	0.117	0.733	0.866	0.378	1.980
Adequate gestational weigh gain			4.604	0.100			
Excessive gestational weigh gain	0.300	0.466	0.414	0.520	1.350	0.541	3.365
Insufficient gestational weigh gain	1.303	0.608	4.595	0.032	3.681	1.118	12.116
Pregnancy-induced hypertension	3.253	0.432	56.826	0.000	25.864	11.102	60.256
Gestational diabetes mellitus	0.693	0.487	2.025	0.155	2.000	0.770	5.194
Migraine worsening	−0.318	0.453	0.493	0.483	0.728	0.300	1.768

B, regression coefficient; S.E., standard error of the coefficient; Wald, Wald chi-square statistic; *p*, statistical significance value; OR, odds ratio; CI, confidence interval.

## Data Availability

The original contributions presented in this study are included in the article. Further inquiries can be directed to the first author (M.L.).
